# Exploring education preferences of Australian women regarding long-term health after hypertensive disorders of pregnancy: a qualitative perspective

**DOI:** 10.1186/s12905-021-01524-w

**Published:** 2021-11-01

**Authors:** Heike Roth, Amanda Henry, Lynne Roberts, Lisa Hanley, Caroline S. E. Homer

**Affiliations:** 1 Faculty of Health, University of Technology Sydney, Sydney, NSW Australia; 2 School of Women’s and Children’s Health, UNSW Medicine, University of New South Wales, Sydney, NSW Australia; 3 Department Women’s and Children’s Health, St George Hospital, Kogarah, Sydney, NSW 2217 Australia; 4 St George Hospital, Sydney, NSW Australia; 5 Burnet Institute, Maternal, Child and Adolescent Health, Melbourne, VIC Australia

**Keywords:** Cardiovascular health, Preeclampsia, Gestational hypertension, Hypertensive disorder of pregnancy, Long-term preventive health education, Women

## Abstract

**Background:**

Hypertensive disorders of pregnancy (HDP) affect 5–10% of pregnant women. Long-term health issues for these women include 2–3 times the risk of heart attacks, stroke and diabetes, starting within 10 years after pregnancy, making long-term health after HDP of major public health importance. Recent studies suggest this knowledge is not being transferred sufficiently to women and how best to transmit this information is not known. This study explored women’s preferred content, format and access to education regarding long-term health after HDP.

**Methods:**

This was a qualitative study and framework analysis was undertaken. Women with a history of HDP who had participated in a survey on long-term health after HDP were invited to participate in this study. During telephone interviews women were asked about preferences and priorities concerning knowledge acquisition around long-term health after HDP.

**Results:**

Thirteen women were interviewed. They indicated that they wanted more detailed information about long-term and modifiable risk factors. Their preference was to receive risk counselling from their healthcare provider (HCP) early after giving birth along with evidence-based, print or web-based information to take home. All women suggested more structured postnatal follow-up, with automated reminders for key appointments. Automated reminders should detail rationale for follow-up, recommended tests and discussion topics to be addressed at the appointment.

**Conclusion:**

Our findings show that most participants wanted information soon after birth with all women wanting information within 12 months post birth, complemented with detailed take-home evidence. Participants indicated preference for structured follow-up via their HCP with automated alerts about the appointment and recommended tests. This evidence can be used to guide the development of education programs for women on health after HDP which may enhance knowledge, preventive health management and more generally improve women’s health trajectories.

**Supplementary Information:**

The online version contains supplementary material available at 10.1186/s12905-021-01524-w.

## Summary

### The known

- Hypertensive disorders of pregnancy (HDP) are linked to increased future health risks
- Studies show that health implications are not sufficiently conveyed to women post HDP
- Research suggests that risk discussions between healthcare providers and women are suboptimal

### The new

- This paper identifies women’s preferences for information regarding content, format and access
- To adequately address their future health, women’s mental health post-HDP needs to be considered

### The implication

- These findings can be used to inform future education and information for women.

## Introduction

Hypertensive disorders of pregnancy (HDP), including preeclampsia (PE), gestational hypertension (GH), and chronic hypertension (CH), affect 5–10% of pregnant women globally [1]. GH, new-onset hypertension only after 20 weeks of pregnancy, may progress to PE but otherwise has good short-term outcomes. CH (hypertension diagnosed pre-pregnancy or in the first half of pregnancy) also may progress to PE, and as a traditional cardiovascular disease (CVD) risk factor already denotes a woman at high longer-term CVD risk. HDP, particularly the multi-system disorder preeclampsia, [2] are a major cause of poor pregnancy outcome leading globally to over 50 000 maternal deaths and 400,000 perinatal deaths each year [3, 4]. Additionally, HDP are associated with substantial longer-term maternal disease. Ischaemic heart disease and stroke, two leading causes of death in women globally, [5] are 2–2.5 times higher after preeclampsia compared to normotensive pregnancies [6–8], with risks of Type 2 diabetes and renal disease also increased [9]. Risks are present within 5–10 years of an affected pregnancy [6, 10, 11] and continue lifelong [8]. GH is also associated with long-term cardiovascular sequelae [8, 12, 13], while as CH is a traditional cardiovascular disease (CVD) risk factor, it already identifies woman as being at higher CVD risk.

Studies conducted globally have found that women have poor knowledge of their increased CVD risks after HDP [14]. Healthcare providers (HCP) often lack the necessary knowledge [14, 15] and when postnatal follow-up care is scheduled, women often do not attend [16]. Many women have poor insight into risk-reducing lifestyle changes and may not engage in making changes, due to family and caregiving responsibilities, lack of knowledge, lack of appropriate and timely follow up, and higher cost of healthier diet [17–20]. Transition from hospital-based to community-based care with general practitioners (GP) is an additional system-level barrier to appropriate post-HDP follow-up, as is lack of health insurance [21].

There is limited understanding of the extent to which HCP conduct appropriate assessment and whether preventive management occurs after HDP. Research suggests women are less likely than men to have their CVD risks fully assessed, or appropriately managed when they are diagnosed [22]. Although international professional associations, including the American Heart Association, recommend asking women about preeclampsia/HDP during CVD risk assessment [23], this has not been formalised in Australian guidelines [24].

As part of broader work on health after HDP, an online survey study assessing knowledge of long-term health and information needs of post-HDP Australian women (n = 266) found that most post-HDP respondents (76%) wanted information 0–6 months postpartum, and from a HCP (80%), key organisations (60%), social media (47%) and brochures/flyers (43%)[25]. However, there is limited understanding of women’s preferences of how best to provide the information [26]. This study explored women’s preferred content, format and access to education regarding long-term health after HDP.

## Methods

### Design

The study design was qualitative and used a framework approach to the analysis. Framework analysis is a data management method to facilitate interpretation of qualitative data [27, 28]. The framework approach has been developed specifically for applied or policy-relevant qualitative research in which the objectives of the investigation are typically set in advance and shaped by the information requirements. It was considered suitable for analysis of our interview data, where comparisons within and between interviews generated themes. The matrix format was useful in the management of the data sets and it facilitated a structured overview of summarised data. The method has five phases that are interlinked, enabling understanding and interpretation of the data and moving from descriptive reporting to conceptual explanation of collected data.

Telephone interviews were chosen because eligible women were geographically diverse, and phone interviews offer greater flexibility in interview scheduling, reduced research costs and faster data collection [29]. Participants have greater anonymity, which may encourage responses to sensitive questions [30] and provide rich data for qualitative analysis [31].

Ethical approval was granted by South-Eastern Sydney Local Health District Human Research Ethics Committee (Ref: 18/POWH/326). The ratification for the University of Technology Sydney was also obtained under ETH18-3061. We confirm that all methods were performed in accordance with the relevant guidelines and regulations associated with the ethics approvals by the above institutions. Informed consent was obtained from all the participants prior to the interview.

### Context

Although geographically large, most Australians (71%) live in major cities of more than 100,000 and < 10% in small towns (less than 10,000) or remote locations [32]. Australia has universal healthcare for citizens and permanent residents, with the federal government being responsible for outpatient facilities such as family doctor/GP while state governments are responsible for public hospitals (where almost all women give birth). Private healthcare is also widely available: approximately 25% of Australian births occur in a private hospital. There are both medically-led and midwifery-led models of care, although as with healthcare services generally these are more restricted in rural and remote regions [33]. Although some women see their GP for pregnancy care, most consult a different care provider for maternity care than their pre-pregnancy and ongoing health care [33, 34].

### Participant recruitment

Participation was open to women who had taken part in a prior survey assessing knowledge of long-term health after HDP [25] which targeted Australian women who either experienced a recent HDP pregnancy or a pregnancy without any serious complications. The previously conducted survey consisted of a custom-created (consumer co-designed) online survey (available in English, Arabic and Mandarin) and was distributed through a targeted convenience sample approach to prior research study participants [35], organisations such as Australian Action on Preeclampsia (AAPEC), maternity consumer groups, through the project’s consumer representative, and social media (Facebook and Twitter) including multicultural networks in order to reach Arabic and Mandarin speaking communities. The 266 participants with (n = 174) or without (n = 92) HDP history were given the option to leave contact details at survey conclusion if interested in follow-up interviews. A total of 61 women who provided their contacts were invited to participate in an interview, and 13 of them accepted the invitation. A study information sheet including consent items were emailed to participants prior to the interviews to allow for reading in their own time, without feeling pressured.

### Data collection

Data collection involved semi-structured telephone interviews, all conducted in English by the same person (HR) from December 2019–January 2020. The interviews were audio recorded with verbal consent of participants and later transcribed. Before commencing interviews, the researcher introduced herself to participants and provided a brief summary of the results from the preceding survey study [25]. Women had the opportunity to ask questions about the study information sheet and consent items. This provided a context for the questions. During the interview, women were asked about preferences and priorities concerning knowledge acquisition around long-term health after HDP (Additional File 1).

### Data analysis

Interviews were transcribed verbatim, then analysed qualitatively using framework analysis [27, 28].

The analysis was an iterative process that involved identifying and developing a thematic framework through an inductive approach [27, 28]. The first author familiarised herself with the raw data by listening to the audio files, reading and re-reading interview transcripts and taking note of preliminary themes by which the data could be examined and referenced in relation to the research aims. These were then discussed and debated with other authors. A matrix developed in Microsoft Excel (V16.16.25 for Macintosh) was used to allocate categories to women’s quotes. Relationships between themes were derived from the data, based on the original research aims and linked to previous quantitative findings [27, 28]. There were no deviant categories identified from the data.

The authors include female HCP and a consumer. As a group we acknowledge our own personal values and positions, including our work within the Australian healthcare system and as recipients of maternity care, may impact on the research process and type of data collected [36].

## Results

The 13 participants included 10 women with a history of PE, two women with a history of GH, one woman with CH and superimposed PE. The majority of the participants (n = 12) were Caucasian while one was Aboriginal/Torres Strait Islander. All women who participated in the study had tertiary education, six were aged 26–35 years, another six were aged 36–45 years while one was older than 46 years. Eleven participants had experienced HDP in the three years preceding the interview. The distribution of participants in the study based on age group, ethnicity, education level, relationship status, and recency and type of HDP, HDP was similar to that of the preceding survey (Additional File 2). Average interview length was 28 min (range 16–36 min). Saturation was achieved after 8 interviews as most women were providing similar suggestions and comparable perceptions. However, all 13 women who had agreed to participate were interviewed to honour their commitment and contribution to the research.

The three main categories identified from the interviews were ‘Accessing evidence-based and comprehensive information’, ‘Transitioning care from hospital to community’ and ‘Fostering self-advocacy’ (Additional File 3).

### Accessing evidence-based and comprehensive information

Women were asked about their current understanding of their CVD and diabetes risks. Their responses highlighted a lack of awareness regarding identification of these risks as well as limited knowledge about, or misinterpretation of, the potential risks for their context (post-HDP). Most women were also unsure what they could do to manage or mitigate their risks.

Women wanted post-HDP information to include HDP definitions and pathophysiology. They wanted to know their own long-term risks as well as their children’s risk, including a better understanding of how HDP leads to future health risks. Women also wanted explanations of how the various CVD and metabolic disorders affected the body and which signs and symptoms to seek medical assistance for.

Women wanted information on modifiable risk factors and how they could specifically address these. This included when and how their risks may manifest, what testing would be needed to identify and address these through early intervention, and instructions to assist them with recommended lifestyle changes and risk reduction. Timely information was important, and as stated by one woman:

The more important thing is actually getting at it at a time where it's timely for me, if I can change things about the way I live and those sorts of things, I don't want to find out 10 years later.

It was also clear that processing the HDP pregnancy and birth takes time and needs to be accounted for, both as an important stand-alone issue, and in the context of knowledge transfer around longer-term health. Despite provision of context and interview questions clearly focusing on long-term health, most women repetitively referred back to their pregnancy and HDP diagnosis, birth and the immediate postnatal time, indicating a persistent focus on these events rather than their future health. One woman shared her post-PE experience:

... I was pretty blue, pretty dark time ... Sometimes just have to take two steps, one step forward, two steps backwards kind of thing. I think sleep deprivation doesn't help ... no one really knew what was going on with me. All the unknowns about it were quite shocking and then to find out afterwards that I have a chance of heart disease and things, is quite scary.

Women wanted structured information accessible verbally, in print and as an electronic resource (smartphone application or a website). Most wanted the information to be specific and detailed but also relatable and easy to understand, addressing needs of women with disabilities (visual and auditory) and preferably catering for those from diverse linguistic and cultural backgrounds. Preference was expressed for highly visible and easy to find information, centralised through a website for example.

Participants’ preferred sources of information included women’s HCP, specialised organisations or targeted social media interest groups such as those focussed on premature birth or preeclampsia. Participants suggested push-notifications such as follow-up appointment reminders. Women wanted information through formal pathways, particularly their HCP, rather than relying on their own informal, self-guided internet searches:

I myself would like to access that directly through my doctor, as in my doctor giving that to me. I rely on them to give me that little bit of information when it comes to my medical future.

A pamphlet or other print resource was mentioned by most women as being acceptable, especially for ‘take home’ information after the risk discussion with the HCP. Others wanted to access information electronically through a website with tailored information or to be emailed from their HCP. Women wanted their HCP risk discussion supplemented by links to reliable research articles or practical information. Women stated that smartphone applications would help ensure information was accessible.

Participants viewed social media as potentially facilitating access to information. Some women felt that accessing information through established organisations was also useful and trustworthy, with AAPEC and the Australian Heart Foundation via Facebook being specifically suggested. One woman suggested push notifications or health notices to women through targeted social media groups.

### Transitioning care from hospital to community

Women wanted to know what their long-term medical follow-ups should include in terms of planning, blood tests and other investigations. Referral or clinical handover letters from the maternity HCP to the woman’s nominated community HCP, detailing pregnancy complications and required long-term follow-up, were suggested. Most women wanted follow-up reminders, likening it to the written and SMS reminders sent by Cervical Screening Registers or Gestational Diabetes Register. They found this format practical and said they were more likely to remember to make a follow-up appointment. Participants also reported that women would feel more involved and informed if there were details about the suggested appointment such as tests to be done or topics to be discussed, for example:

If you're developing those risks five to 10 years later, whether you then receive information from the either federal government or state government. Because I have gestational diabetes, I get a letter saying “you're at risk, make sure this year you get your blood tested”. Whether maybe the same type of communication could go out to the [future] hypertensive patient as well?

The risk discussion needs to be timed such that the woman is able to focus on her own health rather than during the early transition to parenthood. Women who experience unexpected outcomes relating to their baby’s birth may not be able to focus on their own health, short or long term, as this woman explained:

I think the timing is really important because when I had my follow up, my daughter was still in hospital. The information that I was processing at the time, probably wasn't great ... My daughter is one now and I kind of think, well now is the time I'm ready to process information about that [own long-term health].

Some felt that their HCPs lacked knowledge about health after HDP and they were therefore unsure whether to trust their advice on addressing modifiable risks. One woman explained her doubts:

I don't feel ... I mean, I could be wrong, maybe she [GP] does know this. I really like her, and she has been on top of a lot of other stuff. But if she doesn't know, I'm 99% sure that she will never say to me, "I'm going to check your lipids," or "I'm going to check you for signs of whatever." If I don't bring it up, I don't think she will.

The narratives showed that a more structured and pro-active referral approach from maternity to community would be useful. Most of the women felt that a structured transition system would enable them to gradually take ownership of the information and advocate for their own health.

### Fostering self-advocacy

Taking ownership of their health and engaging in self-advocacy and accountability regarding their future health were seen as important by all participants. This enabled women to speak up regarding their health and actively seek knowledge to fill their self-perceived knowledge gaps. Although women wanted to, in a first instance, receive relevant health information from their HCP, they also recognised a degree of self-accountability for their long-term health, and that this may require additional effort to inform the GP of their medical history and to request tests. One woman described her approach to managing her own risk opportunistically:

It is then up to the patient to continue that post care with their GP ... when I go to the GP now and even if it's for, I had a fingernail infection. I'm like, "While I am here, he can take my blood pressure" ... I am so mindful to keep an eye on it now.

Another woman described that the responsibility of sharing health related information when changing to a new GP lies with the woman:

… if you move doctors, that's a bit tricky as well, isn't it? Because you sometimes your file doesn't follow with you. It's up to you to tell your doctor.

One woman felt that relaying important health information to her GP was similar to her alerting restaurant staff to the fact she is allergic to a certain food:

I think that would be more, a lot on my responsibility to a point to say to the GP. Like someone declaring they have a food allergy when going to a restaurant ... This is the first thing you need to say to the doctor, just say, I have history of hypertension, can you please check? If she can log on, and she goes here in my record, she'll see the details.

When asked about how they accessed post-HDP information, the two most popular means were through the internet and participation in HDP research. Although the women preferred to obtain this information via their HCP, they considered internet and participation in research enablers to knowledge acquisition. Most women explained they independently searched for information to get some, or a more comprehensive, understanding of their future health outlook after HDP. For example:

I more or less sort of inquired myself really. Because I think I was just googling. I mean the doctors just said high blood pressure and then that's all, it didn't really go any further than that ... I started googling and then I found out just through myself that yes it can be the cardiovascular heart disease, stroke...it would probably have been good that that was discussed.

Women felt underinformed and wanted more insight and understanding about their pregnancy diagnosis and the associated long-term health impact. This motivated their self-initiated pursuit of information and health advice. As a result, women expressed feelings of being ‘left to their own devices’. For some, participating in HDP studies was the only way they had accessed information regarding their health risks. For others, being aware of health-related family history enabled knowledge acquisition. The women suggested the promotion of health messages via awareness campaigns or days such as ‘World Preeclampsia Day’ were added awareness raising opportunities and served as reminders about health after HDP.

## Discussion

Women articulated their preferred content, format of, and access to, educational material relating to health after HDP. Three main categories were identified: ‘Accessing evidence-based and comprehensive information’, ‘Transitioning care from hospital to community’ and ‘Fostering self-advocacy’.

Experiencing HDP can mean unexpected pregnancy, birth and postnatal outcomes which makes receiving information difficult in the immediate time following such events. The finding suggests a need for appropriate guidance on the timing and format of information provision. Findings from the survey preceding these interviews showed that women preferred receiving information about long-term health 0–6 months postpartum, from a HCP, key organisations, social media and brochures/flyer [25]. The interview findings support these results from the survey and identified additional reasons for these preferences. Participants requested more detailed information regarding their long-term health and how to address any modifiable risks, with their preferred information source being HCP consultation, supported by relevant print or online information for access whenever it suited them. These preferences are similar to findings from previous studies [14] [37].

A study in the USA [19] reports similar findings regarding preferred educational content, such as effects on children and awareness of signs and symptoms of conditions women are at higher risk of, which suggests some common international needs and preferences. In addition, our preceding survey study [25] and Seely et al. [38] found that in Australia and the USA respectively, women do not receive desired information from their HCPs and therefore resort to internet searches. Like this interview study and the preceding survey study [25], Seely et al. in the USA also found that women are not receiving desired information via their HCP and resort to web-based searches. Frustration regarding lack of available information post-HDP was expressed by women in both studies [38], with women emphasising a need for multiple information formats including print, online or mobile application.

In our study, as addressed under ‘Accessing evidence-based and comprehensive information’, women were clearly still affected by the unexpected outcomes of their HDP pregnancy and birth, and often focused on their immediate health and the next pregnancy rather than long-term effects of HDP. Similar to Brown et al.’s study on post-PE health [18], the interviewer of our study had to ask participants to re-direct their focus on their long-term health whilst addressing the questions. Other studies have also found that psychological aspects of experiencing HDP are an important barrier when addressing post-HDP knowledge transmission [17, 38–40], and that women need to process the shock after experiencing severe PE before they can address lifestyle changes [41]. This suggests that psychological sequelae post-HDP need explicit acknowledgment by HCPs and in education materials in order to facilitate support-seeking among women if required before they can process and act on new, longer-term health information.

Women wanted more information delivered to them by their HCP. However, previous studies show that women’s post-HDP knowledge is low or inexistent [14, 25], HCP have suboptimal knowledge [14, 15], and that HCP address women’s direct questions but do not generally raise the topic of post-HDP risks [39]. In view of improved preventive care, these patterns underscore a need for education in both, HCP and post-HDP women. Effective management of their own health may also be hindered if the woman’s knowledge and perception of risk is not in line with actual risk [42]. However, in order to identify risk and establish a preventive approach, CVD primary prevention also requires accurate risk assessment by HCP [43]. The findings suggest that in order to effectively communicate future health risks a number of elements relating to the woman as well as the relay of health information need to be considered. Both, this study and previously published research on the topic [14] demonstrate that factors affecting uptake and understanding of information such as the recency of a woman’s HDP, her health literacy and personal health beliefs need to taken into account. The manner in which health information is relayed, the language used to share it and the presentation are also important considerations for effective communication about future health risks [44, 45]. In general health risk terms, when HCP present the individual risk rather than general population risk better results are obtained in proposed interventions, which requires the HCP to have effective communication skills, adequate knowledge and the ability to calculate this risk [46, 47].

### Strengths and limitations of the study

The interview data have been conducted as part of a broader program of work on women and HCP knowledge and educational needs post-HDP, which is an overall strength as it provides various perspectives and insights. Whilst the focus of the interviews was on gaining insight on women’s educational preferences, our findings also highlight the importance of addressing psychologic sequelae post-HDP. Selection bias is likely, as only women who participated in the preceding online survey were invited to participate in the interviews. Half of the survey participants were recruited from ongoing studies relating to post-HDP research or consumer groups, so knowledge bias is also likely. The women’s level of active engagement in pursuing further contribution to long-term risks as well as their level of motivation to participate in this study, further contribute to knowledge bias.

Although the distribution of women with CH, GH or PE from the preceding survey [25] was similar in the post-survey interviews, the findings may not be representative of all women in Australia. The interviewed women also had a clear understanding of which HDP they experienced, and by the time of the interview had at least some knowledge of their risk factors. If recruitment had been open to a wider population, the findings may have been more diverse in nature. This can be considered a strength given their lived experience and interest in contributing to positive change in this area of health. Despite the preceding online survey being available in Arabic and Mandarin as well as English, neither the survey nor the subsequent interview findings addressed in this paper were adequately reflective of Australia’s cultural diversity. Considerations of the views of younger women (under 26 years of age) and those residing in different contexts (such as urban and rural areas) with different needs regarding post-HDP access to information and services could provide additional insights in preferences pertaining to educational materials. Once education is established it should be piloted to a culturally and age diverse group in order to then cater for a more proportionally balanced, post-HDP demographic.

### Practice implications

Whilst many studies suggest that a first line of intervention to improve health outcomes in women is to inform them of their risk and their CVD screening and prevention options, few studies provide recommendations on what the education should look like [14, 38]. The findings from this interview study, combined with the preceding online survey findings, were intended to inform the implementation of a pilot study, including website and app for women, educational material and website availability for their GP to assist with hospital-to-community transition postpartum, and reminder notifications for women to see their GP for post-HDP care. The findings of this study could inform strategies to address persistent knowledge-to-practice-gaps regarding improving women’s cardiovascular health after HDP. Women’s access to tailored, structured education may in turn contribute to informed choices women make regarding their lifestyle and follow-up care medium to long-term, potentially positively altering a women’s health trajectory.

## Conclusion

Our findings show that women with a lived experience of HDP prefer to receive information from their HCP soon after birth, complemented with information to take home or access later. The participants suggested that ongoing structured follow-up via their HCP including automated reminders, alerting them to follow-up appointments and recommended tests to be conducted may improve knowledge levels and action on change. Psychologic sequelae post-HDP and especially after PE need to be acknowledged in post-HDP risk discussions and addressed in education materials as this will help women access further support as they negotiate their health journey post-HDP. Whilst information alone does not equate to action on change, it is an important ingredient in the approach for change.

## Supplementary Information

**Additional File 1:** Post-survey interview guide for women with a history of hypertensive disorder of pregnancy.

**Additional File 2:** Demographics in numbers and proportions of survey versus interview participants.

**Additional File 3:** Thematic table illustrating interview findings.

## Data Availability

The datasets used and/or analysed during the current study are available from the corresponding author on reasonable request.

## Abbreviations

AAPEC: Australian Action on Preeclampsia
CH: Chronic hypertension worsening in pregnancy and/or with superimposed preeclampsia
CVD: Cardiovascular disease
GH: Gestational hypertension
GP: General practitioner
HCP: Healthcare provider
HDP: Hypertensive disorder of pregnancy
PE: Preeclampsia

## Declarations

### Transparency statement

The article is the authors’ original work, has not been published and is not under consideration for publication elsewhere. All authors have reviewed and approved the final manuscript. The manuscript is an honest, accurate, and transparent account of the study being reported, no important aspects of the study have been omitted. The manuscript presents partial results from Heike Roth’s Ph.D. research. The project is supervised by Caroline Homer and Amanda Henry.
