# Supplementary material for: Exploring education preferences of Australian women regarding long-term health after hypertensive disorders of pregnancy: a qualitative perspective
Source: BMC Womens Health. 2021 Nov 1;21:384. doi: 10.1186/s12905-021-01524-w (PMC8561910; doi:10.1186/s12905-021-01524-w)
Supplement: Supplementary file 1 — Additional File 1: Post-survey interview guide for women with a history of hypertensive disorder of pregnancy. [file 12905_2021_1524_MOESM1_ESM.pdf]

Additional Files

Additional File 1: Post-survey interview guide for women with a history of hypertensive disorder of pregnancy

1.	Having had high blood pressure during one of your pregnancies, what is your understanding of your future risks of health issues?
2.	If you think back to the time of or soon after giving birth to your baby, there are a couple of questions I'd like you to comment on if possible:<br>• what information did you find helpful knowing early on?<br>• In hindsight, is there anything about your future risk or how to lower the risk you have since found out, that would have been helpful to have known earlier - for example when diagnosed or around the time of birth and early follow up?
3.	Could you describe to me your ideal sequence of events with regards to your follow up after a pregnancy and birth complicated by blood pressure? How would you like to have your health followed up?
4.	What else would you, and maybe other women, who get high blood pressure in pregnancy, like to know about future health after a pregnancy complicated with blood pressure problems in pregnancy?
5.	What sort of information would you find essential and also helpful?<br>• How would you like this information presented? What would it look like?<br>• How/Where would you like to access it from?
6.	Discussing your history of high blood pressure in pregnancy with your health care provider (doctor, nurse, midwife) is important. What do you think may be helpful to assist women with this history during their conversation with a healthcare provider?
7.	Some women have suggested the doctor or nurse also talk about any health issues for you when you have your baby checked at the clinic or have vaccinations. How do you think this would work?
8.	Studies show that often women do not attend follow up appointments with their doctors, due to other priorities of being a new parent. How can we improve the ability of women to be able to get to these appointments?
9.	Is there anything else you would like to tell us about management and education after PE or GH that you feel is important for us to consider when creating education?
