# Supplementary material for: Exploring education preferences of Australian women regarding long-term health after hypertensive disorders of pregnancy: a qualitative perspective
Source: BMC Womens Health. 2021 Nov 1;21:384. doi: 10.1186/s12905-021-01524-w (PMC8561910; doi:10.1186/s12905-021-01524-w)
Supplement: Supplementary file 2 — Additional File 2: Demographics in numbers and proportions of survey versus interview participants. [file 12905_2021_1524_MOESM2_ESM.docx]

Additional File 2: Demographics in Numbers and proportions of survey versus interview participants

|  | Survey  Total  (HDP only)  n(%) | | Interview  Total  n(%) |
| --- | --- | --- | --- |
| Total n | | **174 (100)** | **13 (100)** |
| Age | | | |
| *18-25* | | 10 (6) | - |
| *26-35* | | 81 (47) | 6 (46) |
| *36-45* | | 76 (44) | 6 (46) |
| *46+* | | 7 (4) | 1 (8) |
| Ethnicity | | | |
| *Caucasian* | | 165 (95) | 12 (92) |
| *Asian* | | 5 (3) | - |
| *Aboriginal and Torres Strait Islander* | | 1 (1) | 1 (8) |
| *Other** | | 3 (2) | - |
| Highest Educational attainment | | | |
| *Secondary School* | | 23 (13) | - |
| *Diploma/Trade Certificate* | | 60 (35) | 3 (23) |
| *University Degree* | | 90 (52) | 10 (77) |
| *Prefer not to answer* | | 1 (1) | - |
| Relationship Status | | | |
| *In a relationship* | | 162 (93) | 13 (100) |
| *Not in a relationship* | | 11 (6) | - |
| *Prefer not to answer* | | 1 (1) | - |
| Last HDP | |  |  |
| *under 3 years ago* | | 123 (71) | 11 (85) |
| *over 3 years ago* | | 51 (29) | 2 (15) |
| Type of HDP | |  |  |
| *PE* | | 143 (82) | 10 (78) |
| *GH* | | 15 (9) | 2 (15) |
| *CH* | | 16 (9) | 1 (8) |

*Other: Polynesian or Maori (n=1) mixed ethnicity (n=2)

PE = preeclampsia GH = gestational hypertension CH = chronic hypertension, worsening in pregnancy and/or superimposed preeclampsia
