# Supplementary material for: Exploring education preferences of Australian women regarding long-term health after hypertensive disorders of pregnancy: a qualitative perspective
Source: BMC Womens Health. 2021 Nov 1;21:384. doi: 10.1186/s12905-021-01524-w (PMC8561910; doi:10.1186/s12905-021-01524-w)
Supplement: Supplementary file 3 — Additional File 3: Thematic table illustrating interview findings. [file 12905_2021_1524_MOESM3_ESM.docx]

Additional File 3: Thematic table illustrating interview findings

| **Research aims** | **Thematic framework/index** | **Category allocated**  (main category **X** and x if also linked to another category) | | |
| --- | --- | --- | --- | --- |
|  |  | Accessing evidence-based and comprehensive information | Transitioning care from hospital to community | Fostering self-advocacy |
| **Content** | - What is HDP? / Definition of HDP? | **X** |  | x |
|  | - How will HDP affect my child? | **X** | x | x |
|  | - At what age/as of when will it affect my health? | **X** | x | x |
|  | - Explanation of the pathophysiology of HDP | **X** |  | x |
|  | - How can I mitigate risk? How can I reduce the risk? | **X** | x | x |
|  | - What is the recommended long-term follow-up? What do I need to have checked, how often and how long for? | **X** | x | x |
|  | - What are the long-term risks? | **X** | x | x |
|  | - What are the short-term risks? | **X** | x | x |
|  | - What are the signs and symptoms of long-term risks? When do I seek medical assistance? | **X** | x | x |
|  | - Information on medication safety | **X** |  | x |
|  | - Lack of knowledge/confusion displayed by interviewee | **X** |  | x |
| **Format** | - Verbally from healthcare provider supported by information to take home | **X** |  |  |
|  | - Structure, a program ready to follow and a recommended follow-up mapped out |  | **X** |  |
|  | - Reminders like the Cervical Screening Registers or Gestational Diabetes Register notifications |  | **X** |  |
|  | - Create a register like the diabetes register that I joined |  | **X** |  |
|  | - Posters at the GP/Letter in the mail/ Something I can put on the fridge | **X** | x |  |
|  | - Information in different languages including visual and auditory disability | **X** |  | x |
|  | - Easy access | **X** |  |  |
|  | - A website/A smartphone application /A brochure/ t research papers to read | **X** |  | x |
|  | - Electronic so I can store it and access when needed | **X** |  | x |
| **Access** | - Information from the healthcare provider | **X** | x |  |
|  | - Push notifications / health advice- from GP or from a register (like Cervical Screening Registers or Gestational Diabetes Register) or from organisations (email, text or letter) |  | **X** | x |
|  | - Via organisations like Heart Foundation or Australian Action on Preeclampsia | **X** | x | x |
|  | - A website/A smartphone application /A brochure/ t research papers to read | **X** |  | x |
|  | - Research projects I can participate in | **X** | x | x |
|  | - Awareness day | x |  | **X** |
|  | - Social media (e.g. Facebook) - targeted interest groups (e.g. preeclampsia, premature birth) | **X** |  | x |
| **Enablers** | - Knowledgeable healthcare provider | x | **X** |  |
|  | - If my own knowledge is good it will enhance conversation with healthcare provider and my search for information | x | x | **X** |
|  | - I am in charge of my health and should be proactive in my health approach and seeking assistance (but I need knowledge for this) |  |  | **X** |
|  | - Hear information repeated often, at all HCP contact points | **X** |  |  |
|  | - The right timing for the risk discussion |  | **X** |  |
|  | - The right help (the right HCP or service to assist with mitigating risk e.g dietician) |  | **X** | x |
|  | - The specialist writing a letter to my GP | x | **X** |  |
|  | - To have a plan of care - what is the plan - what now? | **X** | x |  |
|  | - Have something written down by my healthcare provider about my HDP | **X** | x | x |
|  | - Follow up more structured like post-diabetes |  | **X** |  |
| **Barriers** | - Psychological sequelae - identified and stated by the women or demonstrated by their answers (e.g. recurrent focus on birth, diagnosis, why? how? what? Rather than looking into future health) | **X** | x | x |
|  | - Does my healthcare provider know about this topic? Do I trust my healthcare provider? | x | **X** | x |
|  | - I should not have to explain this to my GP - What if I don’t explain it correctly to the GP, will they believe me? - I feel weird/intimidated informing my GP of my health risks, I would not bring it up |  | **X** | x |
|  | - Need more information, what happened, no one really explained or maybe I didn’t understand? | **X** | x | x |
